# ASO Author Reflections: What is the Role of Time Interval Between Preoperative Chemotherapy and Surgery in Locally Advanced Pancreatic Cancer?

**DOI:** 10.1245/s10434-026-19507-2

**Published:** 2026-03-31

**Authors:** Marionna Cathomas, Martin Loos

**Affiliations:** Department of General, Visceral and Transplant Surgery, Heidelberg University Hospital, Heidelberg, Germany

## Past

In recent years, multimodal treatment strategies combining preoperative chemotherapy followed by surgery have been established in locally advanced pancreatic cancer (LAPC).^1^ Tumor downstaging achieved through preoperative chemotherapy enables an increasing proportion of patients with LAPC to undergo surgical resection, which remains the main determinant of overall survival.^2^

Currently, clear evidence regarding the optimal time interval between preoperative chemotherapy and surgery is lacking. However, data from other malignancies suggest that this interval may significantly influence oncological the outcome.^3^

## Present

In this retrospective study, patients undergoing pancreatic resection following preoperative chemotherapy were included.^4^ For the final analysis, the exact date of the last chemotherapy cycle was required. Patients were therefore stratified according to the time interval between the last cycle of preoperative chemotherapy and surgery.

The analysis revealed that patients with an extended time interval of ≥ 4 weeks showed improved overall survival, both from the time of LAPC diagnosis (20.7 versus 29.5 months; *p* = 0.019) and from the time of surgery (16.1 vs. 23.1 months; *p* = 0.024). Considering that the effect of preoperative chemotherapy may diminish over time, further statistical analyses were performed to determine an optimal time window, which was found to be between 5 and 8 weeks.^4^

## Future

The results of the present study highlight the importance of the time interval between preoperative chemotherapy and surgery. These findings should be confirmed in a prospective, randomized controlled trial. Extending the time interval represents a simple strategy that may improve oncological outcomes.
